# Modelling the joint impact of early-life interventions on adult health: an illustrative example of multiple long-term conditions with role limitations in midlife using the 1970 British Cohort Study (BCS70)

**DOI:** 10.1186/s12916-025-04467-3

**Published:** 2025-11-18

**Authors:** Sebastian Stannard, Ann Berrington, Nida Ziauddeen, Simon D. S. Fraser, Shantini Paranjothy, Rhiannon K. Owen, Nisreen A. Alwan

**Affiliations:** 1 School of Primary Care, Population Sciences and Medical Education, Faculty of Medicine, University of Southampton, Southampton, UK; 2 NIHR Applied Research Collaboration Wessex, Southampton, UK; 3 School of Economic, Social and Political Sciences, University of Southampton, Southampton, UK; 4 NIHR Southampton Biomedical Research Centre, University of Southampton and University Hospital Southampton NHS Foundation Trust, Southampton, UK; 5 School of Medicine, Medical Sciences and Nutrition, University of Aberdeen, Aberdeen, UK; 6 Population Data Science, Faculty of Medicine, Health & Life Science, Swansea University Medical School, Swansea University, Swansea, UK; 7 University Hospital Southampton NHS Foundation Trust, Southampton, UK

**Keywords:** Multimorbidity, Childhood, Prevention, Intervention, Long-term conditions, Early life

## Abstract

**Background:**

Evidence on how policy interventions early in childhood can prevent or delay multiple long-term conditions (MLTCs) is limited. We modelled prevention scenarios using five early-life domains on the outcome of MLTCs with role-limitation using effectiveness data of combined real-life early interventions.

**Methods:**

Our study sample was 6201 participants in the 1970 British Cohort Study. The outcome was MLTCs with role-limitation (i.e. impacting everyday life functioning) as reported by participants at age 46. We constructed adversity scores within early-life domains (from prenatal to age 10) including prenatal to birth, developmental attributes, education, socioeconomic factors and family environment and used adjusted multivariable logistic regression to examine their relationship with the outcome. We generated adjusted population attribution fractions to estimate the reduction in outcome risk if cohort members reduced their adversity scores. Using effect estimates on early-life exposures from evaluations of real-life interventions including Family Hubs, the Family Nurse Partnership and the teenage pregnancy prevention framework, we calculated the absolute reduction in the outcome risk had cohort members been exposed to all three interventions.

**Results:**

Reducing early life adversity scores from 3 + to 1, from 3 + to 0, from 2 to 0 in the developmental attributes domain and from 3 + to 2, from 3 + to 0, from 1 to 0 in the prenatal to birth domain, lowered the outcome risk. For the developmental attributes domain, the combined effect of the interventions could result in a 0.5% reduction in MLTCs with role limitations for those with a domain adversity score of 3 +. For the prenatal-birth domain, the combined effect of the interventions could result in a 11.5% and 2.5% reduction in MLTCs with role limitations for those with a domain adversity score of 3 + and 1, respectively.

**Conclusions:**

Interventions during pregnancy, the postnatal period and childhood may reduce MLTC risk in midlife.

**Supplementary Information:**

The online version contains supplementary material available at 10.1186/s12916-025-04467-3.

## Background

Multimorbidity or multiple-long term conditions (MLTCs) is commonly defined as living with two or more long-term conditions (LTCs). Having increased in prevalence over the last 20 years in most countries, MLTCs are having a major impact on health and social care systems and people’s lives [1–4]. MLTCs often occurs earlier in the lifecourse among people from more socioeconomically and demographically disadvantaged backgrounds [5]. MLTCs are no longer a condition that is characterised by older age as a substantial proportion of people living with MLTCs are under 65 [5]. Wider social determinants of health such as family structure, education, housing, neighbourhood environment and work influence MLTC risk through multiple mechanistic pathways across the lifecourse [6]. Despite this, there has been little evidence on the combined influence of early-life determinants on MLTCs.

Of the little evidence available, research has often used a life course framework to demonstrate that certain early-life characteristics are associated with MLTCs [7–15]. These include parental social class [7], childhood body mass index (BMI) [7], childhood cognitive ability [7], child behaviour [7], childhood illnesses [8], birthweight [7, 9], individuals born to mothers under the age of 25 [9], maternal BMI [9], those with rapid growth in height and weight from birth until age 11 [9] and those affected by wartime separation [9]. Other research has found that early childhood conditions including parental socioeconomic status [9–14], poor childhood health [10, 14], child maltreatment [15], child adversity including abuse and neglect [12], negative caregiver’s characteristics [12], food restriction [14], child labour [14] and stressful life events [14] were associated with MLTCs across the adult life course.

However, a limitation of this existing research is that it often focussed on single exposure-MLTCs relationships, potentially to reduce statistical complexity, or to focus policy attention onto a specific aspect. In reality, however, children are exposed to combinations of risk factors across multiple early-life domains. In previous research, through a literature and policy scoping review and with support from members of the public, we identified and conceptualised 12 life course domains for future MLTC risk [16]. This work helped to identify the importance of considering multiple childhood exposures, and not only the effect of individual variables that form the components of early-life domains for three reasons. First, conceptualising exposures within wider early-life domains provides a better reflection of the true childhood conditions in which the individual grew up. Second, a combined domain adversity score avoids performing multiple statistical testing of each individual component in relation to the study outcome. Third, consideration of related exposures within domains may better inform pragmatic interventions and policy change in childhood, as single exposure change at one point in time is unlikely to substantially change the trajectories of MLTCs. It is also likely that interventions will target multiple aspects within a domain, as opposed to single exposures or factors.

It is also important that research begin to move away from using a simple count of the number of LTCs experienced towards a more complex understanding of MLTCs, including a consideration of both burden and complexity within the context of MLTCs. In turn, developing a greater understanding of the effect on MLTCs on people’s lives and ways in which living with MLTCs is ‘burdensome’ for people. For example, a recent qualitative evidence synthesis of the experience of living with multiple long-term conditions identified the multifaceted nature of the impact on many aspects of life [17].

The aim of this paper was to model prevention scenarios using five early-life domains on the outcome of MLTCs with role-limitation (i.e. impacting everyday life functioning) using effectiveness data of real-life early interventions. This paper is framed within a life course health development (LCHD) framework [18, 19] that conceptualises health in adulthood as a dynamic trajectory shaped by cumulative exposures and experiences from conception through adulthood. A LCHD framework emphasises that health and functioning are both determined by the interactions between economic, social, behavioural, biological and genetic factors that change throughout the life course, and are embedded in early-life [18, 20].

Key to this framework is identifying factors that impact life trajectories, defined as an assessment of long-term patterns of stability and change [18, 21, 22], and increasing evidence highlights the critical role of early-life conditions, particularly during sensitive developmental windows, in determining long-term health outcomes. For example, adverse childhood experiences (ACEs), including socioeconomic deprivation, maltreatment and chronic stress, have been shown to disrupt neurodevelopment and physiological regulation, contributing to increased susceptibility to non-communicable diseases such as cardiovascular disease, diabetes and mental illness in later life. Whilst children who experience a ‘positive start’ in childhood are more likely to be healthier in adulthood and achieve higher socioeconomic success [23–25].

The LCHD framework stresses the importance of ‘critical stages’ in a person’s life. These stages are broken down into the prenatal period; birth, neonatal period and infancy; childhood; adolescence; early adulthood; and older adulthood. This paper focuses on three of these critical stages—the prenatal period; birth, neonatal period and infancy; and childhood. We are using a LCHD framework to explain the importance of early intervention and sustained investment in child health and wellbeing as a strategy for reducing health inequalities and preventing chronic disease across the lifespan.

## Methods

This work was conducted as part of the Multidisciplinary Ecosystem to study Lifecourse Determinants and Prevention of Early-onset Burdensome Multimorbidity (MELD-B) research collaboration [26].

### Datasets

We used the 1970 British Cohort Study (BCS70) [27] that has followed 17,196 cohort members born in England, Scotland and Wales in 1 week in 1970; to date, there have been 10 sweeps of data collection—4 in childhood and 6 in adulthood. The outcome was self-reported at age 46. All other variables were reported either at birth, age 10 or age 46.

### Outcome

The outcome was a combined MLTCs and role-limitation variable at age 46. MLTCs were self-reported and defined as the reporting of two or more LTCs. LTCs included asthma, diabetes, cancer, high blood pressure, heart problems, eczema, chronic fatigue syndrome, stomach, bowel or gall bladder conditions, bladder or kidney conditions, liver disease, arthritis, stroke, depression, anxiety, hearing loss in one or both ears, epilepsy, eye conditions—blindness and low vision, eye condition—diabetes associated disease, eye condition—glaucoma, endometriosis, Menieres disease and psoriasis.

Role limitation was ascertained using responses to the 36-Item Short Form Survey [28]. Role-limitation due to physical health was coded from 4 binary (0/1) questions, with lower scores indicating greater functional life limitation as a result of physical health problems in the four week prior to interview. Scores were the mean of the total number of questions answered. Following previous guidance [29], scoring 0 (‘yes’) indicated that physical health conditions had limited the cohort member. Role-limitation due to ‘emotional problems’ was coded from 3 binary (0/1) questions, with lower scores indicating greater functional limitation as a result of ‘emotional problems’ in the four week prior to interview. Scores were the mean of the total number of questions answered. Following previous guidance [29], scoring 0 (‘yes’) indicated that emotional health conditions had limited the cohort member. The term ‘emotional problems’ is taken from the original 36-Item Short Form Survey [30], and relates to mental health [28]. The survey questions for both role limitation variables are included in Additional file 1: S1.

We grouped those who reported role limitations for either a physical health or emotional problem with those who reported MLTCs to create a binary (no/yes) *MLTCs with* *role limitation* variable on the assumption that role limitations for either a physical or emotional health problem were related to the LTCs considered. The comparison group consisted of those with no role limitations and no MLTCs. To ensure our control group was free from those with MLTC or role limitations, we excluded from the analyses presented in this paper those who reported role limitation but no MLTCs (*n* = 324), and those who reported MLTCs with no role limitation (*n* = 2056). As a result, our analytical sample was 6201 participants.

### Exposures of interest

We considered five domains that we have previously conceptualised in relation to the risk of MLTCs [17, 31], and had been found to be important for other comorbidity outcomes at midlife [32, 33]. The conceptualisation of these domains was shaped by a review of existing research evidence and policy, and co-produced with public involvement via two workshops [16].

The five domains are:

1. *Prenatal*, *antenatal*, *neonatal and birth domain* reported in the birth interview focused on the period from conception to the onset of labour, the circumstances and outcomes surrounding a birth, and the period immediately following birth.
2. *Developmental attributes and behaviour domain* reported at age 10 focused on the developmental markers of children relating to cognition, coordination, personality types and behavioural traits.
3. *Child education and academic ability domain* reported at age 10 related to the process of learning and educational achievement, especially in educational settings, and the knowledge an individual gains from these educational institutions.
4. *Socioeconomic factors domain* reported at age 10 included factors relating to differences between individuals or groups of peoples caused mainly by their social and economic situation.
5. *Parental and family environment domain* reported at age 10 incorporated the interactions between children and care givers, parenting styles, parental beliefs, attitudes and discipline and wider family factors such as kin networks.

#### Analytical sample

Our sample is based on those who were present at three sweeps; at birth, age 10 and age 46. To reduce potential bias in the estimates due to missing data, we used multiple imputation. This was conducted by chained equations for missing observations at birth, age 10 and 46 [34]. Fifty imputation cycles were constructed under the missing-at-random assumption [35–37], which has been found to be highly plausible in the British birth cohorts [38]. All variables were included in the imputation process. The outcome was included in the imputed models, but imputed outcome values were not used.

### Statistical analysis

#### Step 1: creating domain adversity scores

Additional file 1: Table S2 identifies the variables within each early life domain. These variables were categorised from a previous data audit and principal component analysis that identified early-life variables from multiple sweeps of data that fitted into the five domains of interest [31]. Each variable was derived into a binary score with a score of one indicating adversity and a score of zero indicating no adversity. For continuous measurements where validated or widely accepted cut-offs were available, we used those to define adversity (i.e., birthweight and Rutter behaviour). However, for variables where no cut-off existed in the literature, we applied a pragmatic approach by using the bottom 10% of the distribution within the cohort. This decision was to identify individuals who were meaningfully disadvantaged relative to the rest of the cohort*.* Within each domain, the binary scores were summed to produce an overall domain adversity score, with a higher score indicating increased adversity. Due to the small sample size with very high adversity scores (4 +), all participants who had an adversity score of three or more were combined into the same category.

#### Step 2: nested regression modelling

Multivariable logistic regression models explored the relationship between domain adversity scores and the outcome of MLTCs with role limitation, and in Additional file 1: Table S3, we provide the variables included in the multivariable logistic regression models by the report of MLTCs with role limitations. Firstly, univariately considered the relationship adjusting for sex (ref: men) and ethnicity (ref: white). Then we included all five domains adversity scores in the same model and controlled for sex and ethnicity to identify the strongest relationships, taking into account the effect of the other domains. In the final model, we included adult factors that are potentially linked to both the exposures and the outcome. These were recorded at age 46 and included: number of days of exercise per week (0/1/2/3/4/5/6/7), highest educational qualification (no qualification/General Certificate of Secondary Education/A or AS level/diploma/degree/higher degree), occupational social class (National Statistics Socio-economic classification—1/2/3/4/5/6/7/8), weekly income, smoking status (non-smoker/smoker), hours spent on a weekday watching television (0/1/2/3/4 +), hours spent on a weekday on the internet (not for work related reasons) (0/1/2/3/4 +), living with a partner (yes/no), index of multiple deprivation quintile (1/2/3/4/5/6/7/8/9/10), self-reported financial difficulty (comfortable/doing okay/just about getting by/finding it difficult/finding it very difficult) and alcohol consumption (Alcohol Use Disorders Identification Test for Primary Care (AUDIT-PC) Classification—No alcohol consumption/unproblematic/problematic).

#### Step 3: calculating population attributable fractions

For the domains that were significantly associated with MLTCs with role limitations in the fully adjusted model (step 2), we calculated population attributable fractions (PAFs) using the ‘punaf’ package in STATA V17 [39, 40]. The attributable fraction described the proportion of outcome prevalence that would not have occurred were the rates of the exposure the same as in a defined reference group. PAFs were estimated immediately following the logistic regression modelling (step 2), using the full analytical sample included in those models. The reference population for the PAFs defined as individuals free from multiple long-term conditions (MLTCs) or role limitation was consistent with the reference group used in the regression analyses (step 2). This approach ensured alignment between the regression modelling and the PAF estimation, allowing for interpretable estimates of the proportion of outcome cases that could be attributable to early life adversity domains.

#### Step 4: identify effect estimates from relevant real-life interventions that map onto domains of interest

For domains analysed in step 3, we conducted a scoping literature review to identify effect estimates from relevant real-life interventions that mapped onto domains of interest. Initially, we focussed on effect estimates of the intervention based on United Kingdom (UK) data; however, if this was not available, we included effect estimates from other countries including the United States of America (USA) and the Netherlands. We reviewed evidence from existing literature of the effectiveness of relevant real-life interventions to demonstrate how the combined implementation of these interventions during the cohort members’ childhood could have reduced domain adversity scores.

Interventions were selected based on several criteria. First, they had to relate to early-life domains that were significantly associated to MLTCs with role limitations in the fully adjusted models. For example, if the prenatal, antenatal, neonatal and birth domain were associated with the outcome, we would focus on interventions targeting pregnancy, birth and the early postnatal period. Similarly, if the educational and academic abilities domain was significant, we would prioritise interventions aimed at improving attainment, attendance and reducing school exclusions. Second, we included only interventions with citable literature that reported effect estimates. This often limited our selection to well-established interventions with long-term follow-up data. Third, we focused on interventions that had been implemented in the UK, even if UK-specific evaluations were not available. Fourth, we prioritised interventions that could, in principle, be rolled out universally across the UK, assuming the absence of structural or economic barriers. Finally, we focused on interventions that the cohort members could not have previously been exposed too given they were not in place in the UK during the cohort members childhood (i.e., prior to 1980).

We focused on three complex interventions implemented in the UK, chosen because they supported the early-life domains we considered in this paper. These interventions included the Family Nurse partnership (FNP) [41] a home-visiting programme for first-time young mothers and families. For the FNP, we focused on evaluations that had estimated the effect of the intervention on outcomes including birthweight, smoking during pregnancy, child behaviour, and child temperament. Family Hubs [42], a place for families to go for face-to-face support and information from a variety of services, such as baby groups, parental classes, support with wellbeing and mental health, access to financial and debt advice and infant feeding advice. For Family Hubs, we concentrated on evaluations that had estimated the effect of the intervention on childhood developmental indicators. Lastly, the UK Teenage Pregnancy Prevention Framework [43] that aims to reduce unplanned pregnancies and support young people in developing healthy relationships through improved education and access to contraception and support. For this intervention, we focused on evaluations that had estimated the effect of the intervention on reducing teenage pregnancy.

#### Step 5: calculating the absolute reduction in outcome risk had cohort members been exposed to the combined effect of interventions identified in step 4

Finally, we modelled the absolute reduction in outcome risk had cohort members been exposed to the combined effect of the interventions highlighted in step 4 (i.e. each cohort member received multiple interventions), assuming these interventions had been administered universally across the BCS70 cohort. To accomplish this, we recalculated the original domain adversity scores (calculated in step 1), taking into account the effect estimates of the relevant interventions identified in step 4. For example, an evaluation of FNP suggested that exposure to FNP increased birthweight by 324 g [44]. Therefore, we recalculated each of the BCS70 cohort members’ birthweights, assuming every cohort member’s birthweight had increased by 324 g. Effect estimates that were only applicable to a subgroup of the BCS70 cohort were applied randomly among the population at risk. For example, an evaluation of FNP suggested that exposure to FNP reduced maternal smoking by 8% [45]. Therefore, a random sample of 8% of mothers who smoked during pregnancy were reclassified as non-smokers. This process was repeated for all variables across the domains of interest, where a relevant effect estimate relevant to the variable(s) of interest could be identified from the literature.

Within each domain, the adversity scores (calculated in step 1) were recalculated accounting for the effect of the intervention on reducing domain adversity scores. This allowed us to identify the total number of cohort members who would have reduced their domain adversity scores (e.g. from 3 + to 2, from 2 to 1, from 1 to 0 and so on) had they been exposed to the combined effect of interventions.

Using the effect estimates from the significant PAF scenarios (step 3) and applying them to the actual number of people (with MLTC with role limitations) who reduced their domain adversity scores as a result of being exposed to the combined effect of all three interventions, we identified the number of cohort members who would no longer report MLTCs with role limitations under hypothetical combined prevention scenarios. In doing so, we calculated the fully adjusted absolute reduction in risk of MLTCs with role limitations among certain domain adversity groups.

We acknowledge that in order to estimate the hypothetical impact of interventions, we were required to simulate scenarios in which selected individuals were randomly assigned to receiving the intervention outcome (e.g. smoking cessation). This random assignment was not intended to reflect real-world patterns of intervention uptake, but rather to model a counterfactual scenario where the intervention is applied universally or at scale. These simulations were then applied to fully adjusted regression and PAF estimates, meaning that the estimated reduction in outcome risk was calculated within the context of models that accounted for a wide range of confounders across childhood and adulthood. We acknowledge that this is a simplified approach and does not capture the complex mechanisms by which individuals may be selected into, or respond to, interventions which was beyond the scope of this paper. However, this method allowed us to explore plausible population-level effects while controlling for confounding in the exposure–outcome relationships.

## Results

### Descriptive results for the outcome

Table 1 includes the distribution of outcomes, as shown the most common single long-term conditions at age 46 included depression (23.9%), anxiety (18.6%) and asthma (11.7%). 32.5% of cohort members reported MLTCs regardless of role limitation status, and 12.4% reported role limitation for either a physical or emotional problem regardless of MLTCs status. Further, 11.8% of cohort members reported MLTCs with role limitations.

**Table 1:** Distribution of outcome variables

Long-term conditions	Yes	Total
Asthma	11.70%	8580
Diabetes	3.80%	8580
Cancer	1.50%	8580
High blood pressure	10.30%	8580
Heart problems	2.90%	8580
Eczema	8.80%	8580
Chronic fatigue syndrome	1.50%	8580
Stomach, bowel or gall bladder conditions	11.20%	8580
Bladder or kidney conditions	3.40%	8580
Liver disease	0.60%	8580
Arthritis	7.60%	8580
Stroke	0.60%	8580
Depression	23.90%	8488
Anxiety	18.60%	8481
Hearing—loss one ears	2.20%	8580
Hearing—loss both ears	1.90%	8580
Epilepsy (convulsion or fits)	1.00%	8580
Eye conditions—blindness and low vision	0.40%	8574
Eye conditions—diabetes associated disease	0.30%	8574
Eye conditions—glaucoma	0.50%	8574
Endometriosis	2.30%	8537
Meniere’s disease	0.10%	8580
Psoriasis	2.40%	8579
0 long-term condition (regardless of role limitation status)	42.70%	8580
1 long-term condition (regardless of role limitation status)	24.80%	8580
2 or more long-term conditions (MLTCs) (regardless of role limitation status)	32.50%	8580
Role limitation for physical health condition (regardless of MLTC status)	7.9%	8580
Role limitation for emotional problems (regardless of role limitation status)	8.4%	8580
Role limitation for either physical health or emotional problem (regardless of role limitation status)	12.4%	8580
MLTCs with role limitation^a^	11.9%	6201^a^

^a^The sample size of this group is smaller because we excluded those who reported role limitation but no MLTCs, and those who reported MLTCs with no role limitation from the control group

#### Step 1: creating domain adversity scores

**Fig. 1:**
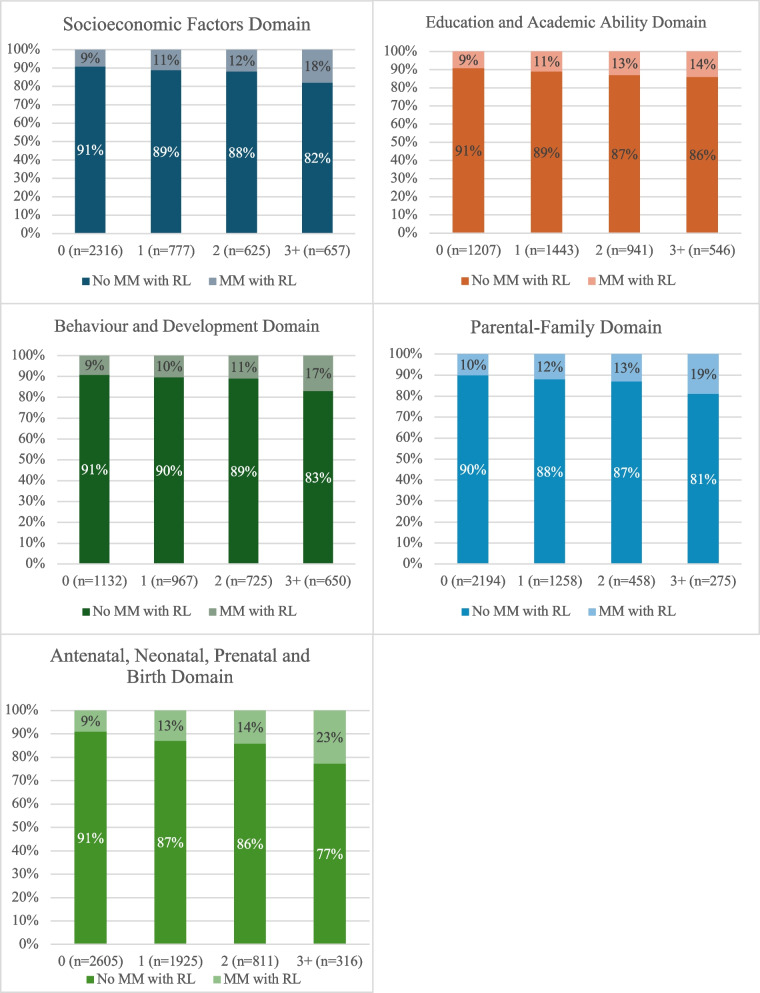
MLTCs with role limitations prevalence at age 46 by domain adversity scores

Figure 1 displays the proportion of cohort members who reported MLTCs with role limitations at age 46 according to the five early-life domain adversity scores. The overall trend was an increasing percentage frequency of MLTCs with role limitations with greater domain adversity scores, with a particularly high prevalence observed amongst those with the highest domain adversity scores (3 +) across the five domains.

#### Step 2: nested regression modelling

Table 2 presents odds ratios from the multivariable logistic regression of MLTCs with role limitation according to domain adversity scores adjusting for sex and ethnicity (model 1), then model 1 plus the other domains (model 2) and finally model 2 plus adult factors (model 3). As shown, adjusting for sex and ethnicity (model 1), there was a significant relationship across domains demonstrating that with an increase in domain adversity scores the odds of reporting MLTCs with role limitations increased, compared to a domain adversity score of zero. There was a particularly strong relationship between cohort members with an adversity score 3 + and odds of MLTCs with role limitations across domains.

**Table 2:** Odds ratios of five childhood domain and MLTCs with role limitation. Adjusting for sex and ethnicity (model 1), the other domains (model 2) and adult factors (model 3)

***N*** = 6201 (50 imputations)		Model 1: Adjusting for sex and ethnicity		Model 2: Adjusting for other domains, sex and ethnicity		Model 3: Adjusting for other domains, sex, ethnicity and adult factors^a^	
	Score	OR	95% CI	OR	95% CI	OR	95% CI
Socioeconomic factors—ref: 0	1	**1.28**	**1.01**–**1.61**	1.13	0.89–1.44	1.01	0.85–1.47
	2	**1.43**	**1.12**–**1.84**	1.18	0.90–1.53	0.94	0.68–1.41
	3 +	**2.20**	**1.76**–**2.75**	**1.55**	**1.19**–**2.02**	0.92	0.67–1.23
Education and academic ability—ref: 0	1	**1.30**	**1.03**–**1.66**	1.22	0.96–1.56	1.12	0.85–1.47
	2	**1.51**	**1.16**–**1.97**	**1.31**	**1.01**–**1.71**	1.04	0.76–1.41
	3 +	**1.85**	**1.43**–**2.39**	**1.31**	**1.00**–**1.72**	0.89	0.64–1.23
Development and behaviour domain—ref: 0	1	**1.29**	**1.01**–**1.66**	1.22	0.95–1.56	1.14	0.87–1.49
	2	**1.56**	**1.23**–**2.00**	**1.41**	**1.10**–**1.81**	1.32	0.99–1.74
	3 +	**2.44**	**1.93**–**3.09**	**2.09**	**1.63**–**2.67**	**1.65**	**1.25**–**2.19**
Parental-family environment—ref: 0	1	**1.36**	**1.13**–**1.65**	**1.23**	**1.01**–**1.49**	1.19	0.96–1.49
	2	**1.42**	**1.10**–**1.86**	1.09	0.82–1.44	0.89	0.64–1.25
	3 +	**2.16**	**1.63**–**2.86**	**1.49**	**1.10**–**2.03**	1.25	0.88–1.80
Prenatal, antenatal, neonatal and birth—ref: 0	1	**1.49**	**1.22**–**1.82**	**1.33**	**1.08**–**1.63**	1.23	0.98–1.58
	2	**1.66**	**1.31**–**2.12**	1.27	0.97–1.64	1.10	0.81–1.49
	3 +	**2.83**	**2.10**–**3.81**	**2.01**	**1.45**–**2.78**	**1.70**	**1.14–2.52**

^a^Highest educational qualification, number of days exercise per week, hours spent on the internet each day, hours spent watching TV each day, occupational social class, alcoholic drink consumption, smoking status, self-perceived financial status, weekly income, IMD quintile and partnership status. Statistically significant results using a 95% level are included in bold

After additionally controlling for all other domains (model 2), those with domain adversity scores of 1 or 2 in the socioeconomic factors domain, those with domain adversity score of 1 in education and academic ability domain or developmental behaviour domain, and those with a domain adversity score of 2 in the parental-family environment domain or prenatal, antenatal, neonatal and birth domain were no longer associated with MLTCs with role limitation. However, for all domains there remained a significant relationship between cohort members with an adversity score of 3 + and odds of MLTCs with role limitations, although odds ratios were reduced. In Additional file 1: Tables S4–8, we demonstrate that the socioeconomic factors domain was an important domain for reducing the effect sizes of the other domains on the outcome. The socioeconomic factors domain particularly reduced the effect sizes of the prenatal, antenatal, neonatal and birth domain and the parental-family environment domain.

In the final model (model 3), we included adult factors at age 46, the full regression model is included in Additional file 1: Table S9. As shown, the inclusion of adult factors attenuated the previously significant relationships between the socioeconomic factors domain, educational and academic ability domain, and parental-family environment domain and odds of MLTCs with role limitations.

However, after adjusting for adult factors, for the prenatal, antenatal, neonatal and birth domain, cohort members with an adversity score of 3 + remained significantly associated with increased odds of reporting MLTCs with role limitation compared to cohort members with a domain score of zero (odds ratio (OR)1.70 95%CI 1.14–2.52). For the development and behaviour domain, cohort members with an adversity score of 3 + remained significantly associated with increased odds of reporting MLTCs with role limitation compared to cohort members with a domain score of zero (OR1.65 95%CI 1.25–2.19).

#### Step 3: calculating population attributable fractions

In the fully adjusted models, two domains remained significantly associated with MLTCs with role limitations—the antenatal, neonatal, prenatal and birth domain and the behaviour and development domain. For those domains we modelled fully adjusted population attributable fractions (PAFs), using the adjusted risk estimates from the logistic regression model (model 3). We present the PAFs scenarios in Table 3.

**Table 3:** Population attributable fractions modelling hypothetical scenarios in the reduction of MLTCs with role limitations for the behaviour and development domain and antenatal, neonatal, prenatal and birth domain. Adjusting for sex, ethnicity, other domains and adult factors

*N*=6201 (50 imputations)				
	Behaviour and development domain		Antenatal, neonatal, prenatal and birth domain	
Scenarios	Percentage reduction	95% CI	Percentage reduction	95% CI
3+ to 2	12.2%	−2.6% - 29.8%	**20.0%**	**2.3% - 34.4%**
3+ to 1	**19.5%**	**6.1% - 29.7%**	15.7%	−2.3% - 29.0%
3+ to 0	**25.8%**	**13.0% - 37.9%**	**24.0%**	**8.0% - 37.2%**
2 to 1	8.8%	−8.0% - 17.3%	−7.7%	−27.3% - 8.8%
2 to 0	**16.6%**	**0.8% - 27.6%**	5.7%	−12.3% - 20.9%
1 to 0	8.5%	−8.2% - 26.8%	**13.0%**	**0.1% - 24.3%**

Statistically significant PAFs using a 95% level are included in bold

For the behaviour and development domain, if cohort members with a score of 3 + moved to a score of 1 or 0, there could be a 19.5% (95%CI 6.1–29.7%) and 25.8% (95%CI 13.0–37.9%) reduction in MLTCs with role limitations amongst cohort members with an adversity score of 3 +, respectively. Within the same domain, if cohort members with a score of 2 moved to a score of 0 there could be a 16.6% (95%CI 0.8–27.6%) reduction in MLTCs with role limitations amongst cohort members with an adversity score of 2.

For the antenatal, neonatal, prenatal and birth domain, if cohort members with a score of 3 + moved to a score of 2 or 0, there could be a 20.0% (95%CI 2.3–34.4%) and 24.0% (95%CI 8.0–37.2%) reduction in MLTCs with role limitations amongst cohort members with an adversity score of 3 +, respectively. Within the same domain, if cohort members with a score of 1 moved to a score of 0 there could be a 13.0% (95%CI 0.1–24.3%) reduction in MLTCs with role limitations amongst cohort members with an adversity score of 1.

#### Step 4: identifying effect estimates from relevant real-life interventions that map onto domains of interest

As previously discussed, we focused on interventions including the FNP [41], Family Hubs [42] and the UK Teenage Pregnancy Prevention Framework [43]. Table 4 includes the variables within each domain that made up the domain adversity scores and how the effect estimates from the three interventions mapped onto these variables. We provide a summary of how the effect estimates were modelled on the variables to analyse the effect of the intervention. We were unable to find effect estimates for interventions addressing either mother’s age at delivery (excluding teenage pregnancy) or parity, and after discussing with the research team it was agreed there were unlikely to be specific policies (within the UK) that target these two factors.

**Table 4:** Mapping effect estimates from real life policy interventions to significant domains

Domain	Variable within domain	Intervention	Effect estimates from evaluations of interventions	How was effect estimate modelled onto original variable of interest
Prenatal, antenatal, neonatal and birth domain	Birthweight	Family nurse partnership [41]	Those exposed to FNP in childhood had a 324 g increase in birthweight [44]	Every cohort member’s birthweight was increased by 324 g
	Smoking	Family nurse partnership [41]	Being part of the FNP led to a 8% reduction in smoking in pregnancy [45]	A random sample of 8% of mothers who smoked during pregnancy were reclassified as not smoking during pregnancy
	Ever had a teenage pregnancy	UK teenage pregnancy prevention framework [43]	The teenage pregnancy prevention framework has led to a reduction in under-18 conception of 62% over the past 18 years through improvements in sexual education and access to contraception and support [46]	A random sample of 62% of mothers with a teenage pregnancy were reclassified as never having a teenage pregnancy
	Mother’s age at delivery	No interventions available	–	–
	Parity	No interventions available	–	–
Development and behaviour domain	Rutter behaviour^a^	Family nurse partnership [41]	For those who were exposed to the FNP there was a 39% reduction in internalising disorders [47]	A random sample of 39% of children who classified as ‘high risk’ on the Rutter behaviour scale^1^ (which includes internalising disorders) were reclassified as ‘low risk’
	Steps walking backwards	Family hubs [42]	Attending Family hubs resulted in a 6% increase in attaining a good level of development early in life [48]	A random sample of 6% of children who had adverse scores for walking backwards (an indication of lower development) were reclassified as no longer having adverse scores
	Child behavioural problems	Family nurse partnership [41]	For those who were exposed to the FNP there was a 55% reduction in the reporting of emotional/behavioural problems [49]	A random sample of 55% of children with a behavioural problem were reclassified as no longer having a behavioural problem
	Child hand control	Family hubs [42]	Attending Family Hubs resulted in a 6% increase in attaining a good level of development early in life [48]	A random sample of 6% of children who had poor hand control (an indication of lower development) were reclassified as having good hand control
	Child temper	Family nurse partnership [41]	For those who were exposed to the FNP there was a 2.6% reduction in aggression score [50]	A random sample of 2.6% of children who were reported as having a temper were reclassified as no longer having a temper
	Child clumsy at games	Family hubs [42]	Attending Family hubs resulted in a 6% increase in the attaining a good level of development early in life [48]	A random sample of 6% of children who were clumsy (an indication of lower development) were reclassified as not being clumsy
	Balance—right leg	Family hubs [42]	Attending Family hubs resulted in a 6% increase in the attaining a good level of development early in life [48]	A random sample of 6% of children who had poor balance (an indication of lower development) were reclassified as having good balance
	Balance—left leg	Family hubs [42]	Attending Family hubs resulted in a 6% increase in the attaining a good level of development early in life [48]	A random sample of 6% of children who had poor balance (an indication of lower development) were reclassified as having good balance

^a^A scale that provides an indication of behaviour difficulties

#### Step 5: calculating the absolute reduction in outcome risk had cohort members been exposed to the combined effect of interventions identified in step 4

The final step was to focus on those cohort members who had reported MLTC with role limitations at age 46, and the significant PAF scenarios identified in Step 3. Using the effect estimates from the significant PAF scenarios and applying them to the actual number of people (with MLTC with role limitations) who reduced their domain adversity scores (as a result of being exposed to the combined effect of the interventions), we calculated the absolute reduction in outcome risk amongst certain domain adversity groups.

### Development and behaviour domain

**Fig. 2:**
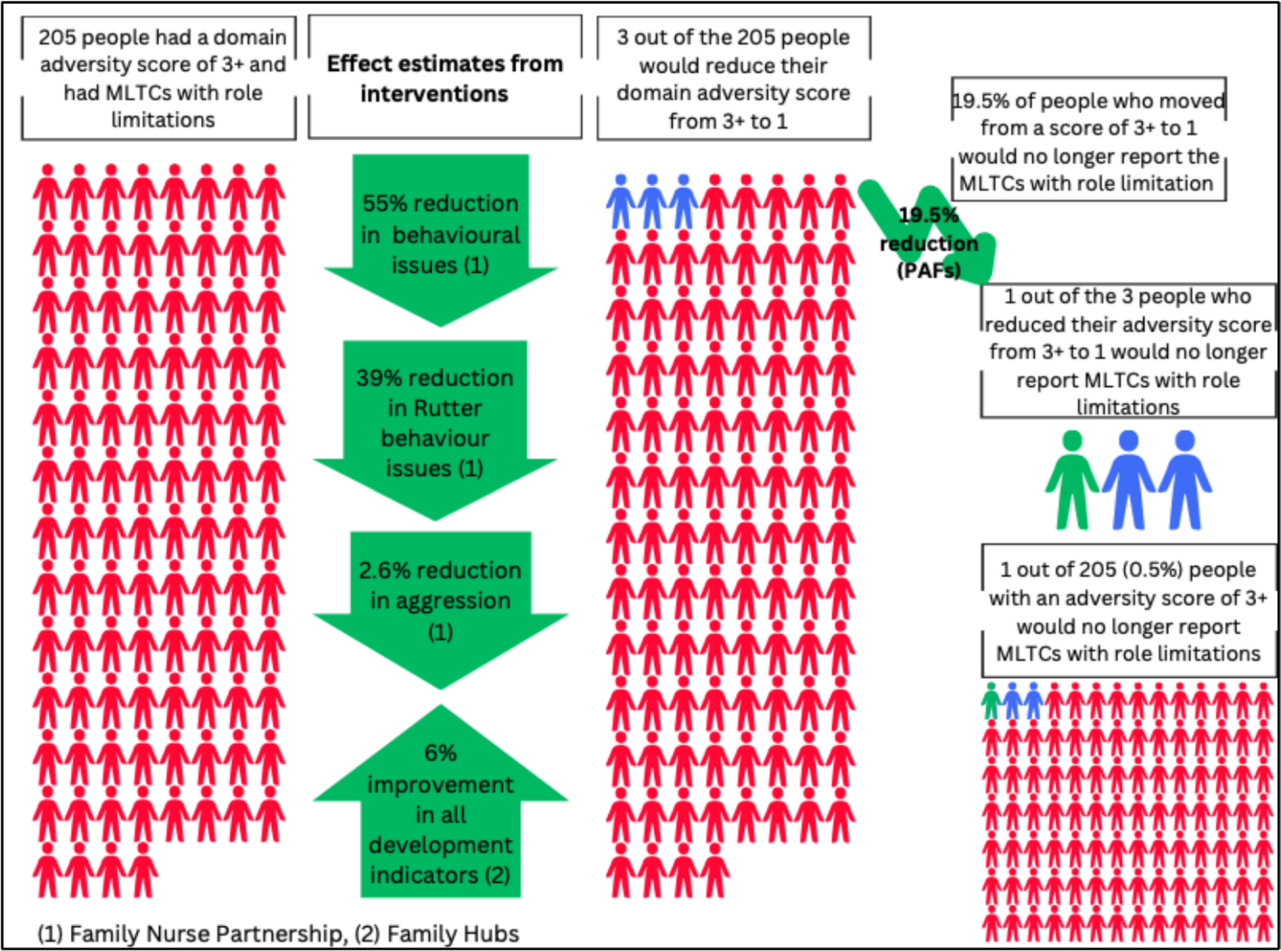
Modelling the fully adjusted changes in adversity scores and subsequent absolute reduction in risk of MLTCs with role limitation, for the significant PAF scenario of moving from a score of 3 + to 1 within the development and behaviour domain

In Fig. 2, and Additional file 1: Figs. S10, S11 we focus on the three significant PAFs scenarios identified in step 3. These included moving from 3 + to 1, from 3 + to 0, and from 2 to 0. As shown, we estimated that three out of 205 (1.6%) cohort members with a domain adversity score of 3 +, and who reported MLTCs with role limitations, would reduce their domain adversity score to 1. One out of 163 (0.9%) cohort members with a domain adversity score of 2, and who had reported MLTCs with role limitations, would reduce their domain adversity score to 0, and less than one person (out of 205) with a domain adversity score of 3 +, and who had reported MLTCs with role limitations, would reduce their domain adversity score to 0.

**Fig. 3:**
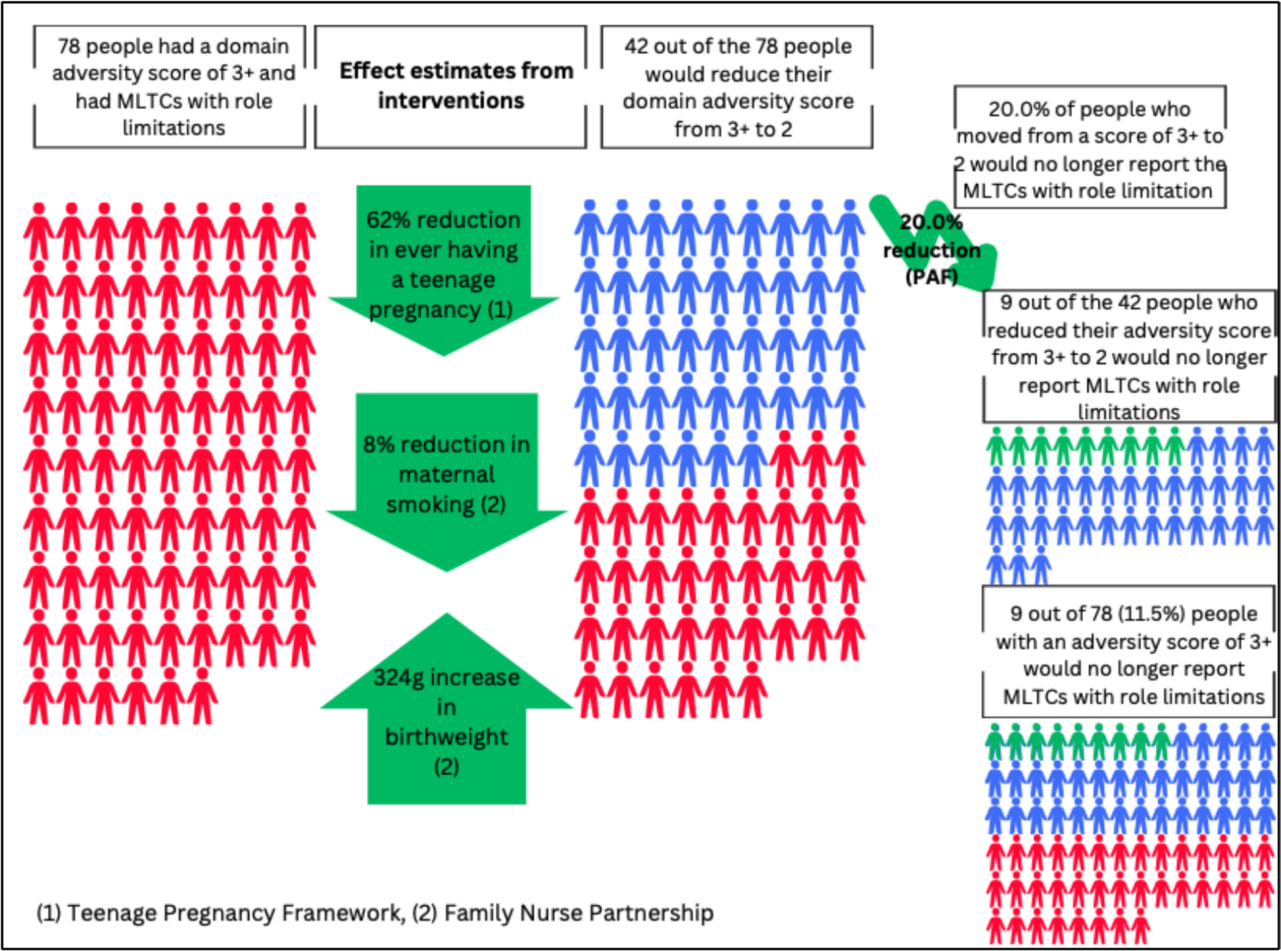
Modelling the fully adjusted changes in adversity scores and subsequent absolute reduction in risk of MLTCs with role limitation, for significant PAF scenarios of moving from 3 + to 2 within the prenatal, antenatal, neonatal and birth domain

**Fig. 4:**
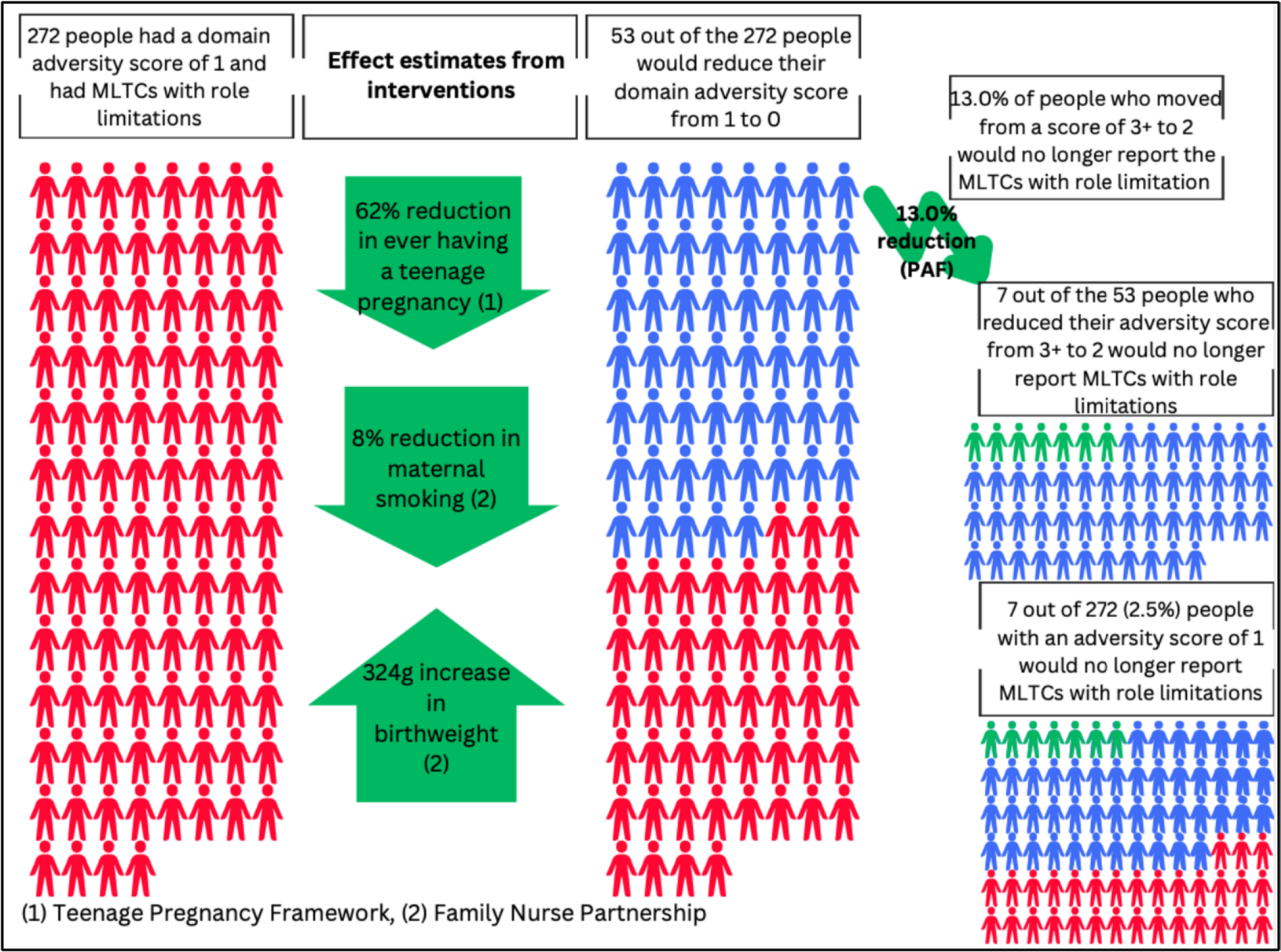
Modelling the fully adjusted changes in adversity scores and subsequent absolute reduction in risk of MLTCs with role limitation, for significant PAF scenarios of moving from 1 to 0 within the prenatal, antenatal, neonatal and birth domain

From the PAFs analysis, we identified that 19.5% of people with reduced adversity scores from 3 + to 1 would no longer have MLTC with role limitation, and applying that 19.5% reduction to the three people who moved from domain adversity score 3 + to domain adversity score 1, we projected that one out of those three cohort members would no longer report MLTCs with role limitations at age 46 (Fig. 2). This represents a 0.5% absolute reduction in the risk of MLTCs with role limitations amongst all cohort members with a domain adversity score of 3 + (Fig. 2). For the other two significant PAFs scenarios, only a very small number of individuals with MLTCs with role limitations attained these PAF scenarios (one person moved from 2 to 0 and less than one person moved from 3 + to 0) (Additional file 1: Figs. S10, S11). Therefore, after applying the percentage reduction in the outcome from the PAF analysis (2 to 0 led to a 16.6% reduction and 3 + to 0 led to a 25.8% reduction), there was no reduction in the risk of MLTCs with role limitations for these groups (Additional file 1: Figs. S10, S11).

### Prenatal, antenatal, neonatal and birth domain

In Figs. 3 and 4 and Additional file 1: Fig. S12, we focus on the three significant PAFs scenarios identified in step 3; these included moving from 3 + to 2, from 3 + to 0, and from 1 to 0. As shown, we estimated that 42 out of 78 (53.8%) cohort members with a domain adversity score of 3 +, and who had reported MLTCs with role limitations, would reduce their domain adversity score to 2 (Fig. 3). 53 out of 272 (19.5%) cohort members with a domain adversity score of 1, and who had reported MLTCs with role limitations, would reduce their domain adversity score to 0 (Fig. 4), and less than one person (out of 78) with a domain adversity score of 3 +, and who had reported MLTCs with role limitations, would reduce their domain adversity score to 0 (Additional file 1: Fig. S12).

From the PAFs analysis, we identified that 20.0% of people who reduced adversity scores from 3 + to 2 would no longer have MLTC with role limitation, and applying that 20.0% reduction to the 42 people who moved from domain adversity score 3 + to domain adversity score 2, we projected that nine out of those 42 cohort members would no longer report MLTCs with role limitations at age 46 (Fig. 3). This represents a 11.5% absolute reduction in the risk of MLTCs with role limitations amongst all cohort members with a domain adversity score of 3 +. For the PAF scenario of moving from a score of 1 to 0, we identified that 13.0% of people would no longer report MLTC with role limitation, and applying that 13.0% reduction to the 53 people who moved from domain adversity score 1 to domain adversity score 0, we projected that seven out of those 53 cohort members would no longer report MLTCs with role limitations at age 46 (Fig. 4). This represents a 2.5% absolute reduction in the risk of MLTCs with role limitations amongst all cohort members with a domain adversity score of 1. The final significant PAF scenario involved moving from 3 + to 0, and if this PAF scenario could be successfully achieved then there would be a 24.0% reduction in the number of people with MLTC with role limitation. However, after modelling the effect of the intervention, less than one person reduced that adversity score from 3 + to 0, and therefore there was no reduction in the risk of MLTCs with role limitations for this group (Additional file 1: Fig. S12). Given, the complexity of this analysis, we have summarised the results in a case study in Table 5.

**Table 5:** Illustrative Case Study: Modelling the Impact of Early-Life Interventions on MLTCs with Role Limitations

CASE STUDY** - **Antenatal, neonatal, and birth domain
Background: Joe is a 46-year-old man with several long-term health conditions that impact his daily life and work. When Joe was born, his mother was 18, she had a previous pregnancy when she was a teenager, and she smoked during her pregnancy with Joe. Joe was born with a low birthweight of 2300g.
Adversity Score and Impact: Due to these factors, Joe had a high adversity score of 3+ within the antenatal, neonatal, and birth domain. This high adversity score increased Joe’s chances of having long-term health conditions by 1.7 times compared to someone with a score of 0, even after considering other factors in childhood and adulthood. Joe is one of 78 people in the BCS70 cohort who had an adversity score of 3+ and were living with multiple long-term health conditions that affect daily life and work at age 46.
Potential Interventions: Our research indicates that if Joe and others with similar scores had been able to reduce their adversity score from 3+ to 2, their chances of having long-term health conditions that impact daily life and work would have decreased by 20%. Therefore, we identified several current interventions that could have improved Joe's adversity score, had these interventions be implemented in the 1970s when Joe was a child. For example, if Joe's mother had received support from programs like the Family Nurse Partnership and Family Hubs, and if the UK had implemented a teenage pregnancy framework in 1970, Joe's birthweight could have increased by 324 g, and Joe’s mother could have been one of 8% of mothers to quit smoking before pregnancy. Joe’s mother could still have been 18 when he was born, and had a previous teenage pregnancy.
Estimated Outcomes: Overall, these interventions could have reduced Joe's adversity score from 3+ to 2, and Joe could have been one of 42 out of 78 people in the BCS70 cohort with a high adversity score who lowered their score to 2. Importantly, 20% (or nine people) out of those 42 people who reduced their adversity score would no longer have multiple long-term health conditions that affect their daily life, and one of those nine people could have been Joe.
Conclusion: In summary, if the 78 people with high adversity scores had received these interventions, nine of them (11.5%) would no longer report long-term health conditions that affect their daily life and work.

## Discussion

Applying data from a national birth cohort, we have established that two early life course domains—the prenatal, antenatal, neonatal and birth domain and the child behaviour and development domain are significantly associated with MLTCs with role limitation after accounting for sex, ethnicity, other early life domains and adult factors that potentially mediate their relationship with the outcome. We found that the socioeconomic factors domain was an important confounder for the other childhood domains, although the domain itself had an attenuated association with the outcome once adult factors had been included in the model. Fully adjusted PAFs demonstrated that reducing adversity scores from 3 + to 1 (adjusted PAF 19.5% 95%CI 6.1–29.7%), from 3 + to 0 (25.8% 95%CI 13.0–37.9%), from 2 to 0 (16.6% 95%CI 0.8–27.6%) in the developmental attributes domain, and from 3 + to 2 (20.0% 95%CI 2.4–34.4%), from 3 + to 0 (24.0% 95%CI 8.0–37.2%), from 1 to 0 (13.0% 95%CI 0.1–24.3%) in the prenatal to birth domain, significantly lowered the outcome risk.

We wanted to quantify our effect in relation to evidence-based real-life interventions. Modelling combined effect estimates from interventions focussing on Family Hubs, FNPs and the UK teenage pregnancy prevention framework, and applying corresponding PAFs, we demonstrated that the combined effect of these intervention would reduce domain adversity scores and subsequently reduce the risk of MLTCs with role limitations at age 46.

For the developmental attributes domain, the combined effect of the intervention would result in a 0.5% absolute reduction in the risk of MLTCs with role limitations for those with a domain adversity score of 3 +. For the antenatal, neonatal and birth domain, the combined effect of the intervention would result in a 11.5% and 2.5% absolute reduction in the risk of MLTCs with role limitations for those with a domain adversity score of 3 + and 1, respectively.

This study modelled hypothetical scenarios based on real-world interventions, applied to the BCS70 birth cohort. We acknowledge that this cohort could differ in important ways from the general population currently receiving these interventions, and these differences may introduce bias that limit the generalisability of our findings. However, our intention was to explore what could potentially have happened if such interventions were implemented in early-life, using a well-characterised cohort to illustrate plausible population-level effects. The findings should therefore be interpreted as exploratory and illustrative, rather than predictive or directly applicable to contemporary populations. However, this approach offers a pragmatic way to inform policy discussions around the potential long-term implications of early-life interventions, especially when direct experimental evidence is unavailable.

Although we modelled the combined effect of multiple interventions, we believe it is feasible that a cohort member could have been exposed to all three interventions. For example, the UK teenage pregnancy framework was a national, population-level initiative aimed at improving access to sexual education and contraception. Similarly, the FNP and Family Hubs are complementary in both timing and scope. The FNP is a structured, home-visiting programme that begins in early pregnancy and continues until the child is aged two. It provides intensive, personalised support to first-time young mothers, focusing on maternal health, parenting skills and child development. Family Hubs, on the other hand, offer a broader, community-based model of support that typically begins after birth and extends into early childhood and beyond. They provide integrated services including parenting support, early education and health services for families with children of all ages up to 18. Because these two programmes target different but overlapping stages of the early life course, it is both feasible and potentially beneficial for families to access them concurrently or sequentially. Their combined implementation reflects a layered approach to early intervention, consistent with a life course perspective.

We acknowledge that different types of early-life domains may vary in their responsiveness to interventions we have considered. For example, the Teenage Pregnancy Framework is specifically focused on the reduction in teenage pregnancy rates and therefore this intervention will only target adversity within prenatal, antenatal, neonatal and birth domain. Whereas both the FNP and Family Hubs target a wider range of adversities in the childhood by supporting families facing challenges such as poverty, poor parental mental health, low educational attainment and social isolation. In addition, the FNP focuses on intensive home visits, promoting healthy parenting and maternal wellbeing, whilst Family Hubs provide integrated, place-based services for families, addressing issues like family conflict, developmental delays and fragmented access to support. Therefore, these interventions are likely to target adversity across all five early-life domains considered in the paper.

Further, our modelling assumed that the effects of individual interventions are additive when applied in combination. However, these interventions have not been evaluated together within a single population sample, and it is possible that overlapping mechanisms could lead to diminished effects. Conversely, given that the three interventions target different stages of the child lifecourse, it is also plausible that their combined impact is underestimated. As such, the estimated effects should be interpreted with caution. Future research should continue to aim to empirically evaluate the joint implementation of multiple early-life interventions to better understand their interactive effects and real-world feasibility.

Previous research has investigated single early-life exposures on the development of MLTCs [7–15]. We quantified the effect of domain adversity scores -rather than individual early-life factors- on a joint MLTC and burden outcome because we recognise that prevention policy choices are rarely feasible or practical for one isolated risk factor. Children’s experiences across a variety of early lifecourse domains are intersecting, and therefore research needs to be able to incorporate information from multiple domains into the same analysis. Conceptualising domains may help to address the early wider determinants of health.

In addition, by considering role limitations, this paper has taken a step towards enhancing MLTC research by moving away from LTC counts towards a more complex understanding of MLTCs. This supports the National Institute for Health and Care Excellence multimorbidity guidance highlighting the need to gain a better understanding of burdensome and complexity within the context of multimorbidity [51].

Our results indicated that even after considering the confounding role of other early-life domains and adult factors, high levels of adversity within the prenatal, antenatal, neonatal and birth domain and behaviour and development domain are associated with MLTCs with role limitations at age 46. This supports previous findings which suggest that the individual early-life factors (included within these domains) such as parental social class [7, 9], birthweight [7, 9], behaviour [7], maternal age [9] and parental socioeconomic status [10–14] are associated with MLTCs. Our work also supports research that utilise a lifecourse perspective to demonstrate that the early-life environment can have a significant impact on multiple dimensions of health across the lifecourse [52, 53].

Our findings underscore the importance of adopting intervention strategies much earlier in the life course. By identifying early-life domains that are significantly associated with the development of MLTCs and role limitations in midlife, our study highlights critical windows of opportunity for preventive action. This aligns with the life course framework, which emphasises how exposures and experiences during early development can have long-term consequences for health and wellbeing. Our results support a shift in focus toward earlier, upstream interventions—particularly those targeting behavioural and developmental conditions at age 10 and the pregnancy period—before health conditions become entrenched. We also suggest that future research and policy should prioritise and continue to invest in scalable, evidence-based interventions that can be implemented during these early-life periods to reduce the long-term burden of MLTCs.

Further, this work also supports the UK healthcare system decision to shift towards a more preventive model of health. The Department of Health and Social Care [54] policy paper on transforming the public health system highlighted the need to focus on prevention and the wider determinants of health, and the 2018 paper on the Public Health Priorities in Scotland [55] that highlighted the importance of investing early in young people’s future as the best form of prevention.

Our results suggested that the interventions had a smaller impact on reducing MLTCs with role limitations for cohort members with adversity within the development and behavioural domain compared to the prenatal, antenatal, neonatal and birth domain. We hypothesise that there are two reasons for this finding. Firstly, the effect estimates of the real-life interventions considered were larger in relation to the variables within the prenatal, antenatal, neonatal and birth domain compared to the development and behaviours domain. Secondly, the significant PAFs scenarios were more extreme for the development and behaviours domain compared to the prenatal, antenatal, neonatal and birth domain. For example, the smallest significant PAF scenario comprised of a reduction in adversity score of 2; however, for the prenatal, antenatal, neonatal and birth domain, the smallest significant PAF scenario comprised of a reduction in adversity score of 1. Consequently, fewer cohort members within the development and behaviours domain achieved these significant PAFs reduction scenarios having being exposed to the interventions.

We found that the socioeconomic factors domain was an important confounder for the other early-life domains supporting research that emphasises the importance of childhood socioeconomic factors for a wide array of health, cognitive and socioemotional outcomes in children [56–59]. Although we were unable to conduct subgroup analyses due to sample size limitations, we also acknowledge that socioeconomic factors may influence the effectiveness of interventions. Individuals from different socioeconomic backgrounds may have varying levels of access, engagement or responsiveness to interventions, which could result in differential benefits across groups. However, to account for this we adjust for a range of socioeconomic factors in both childhood and adulthood, and therefore this approach maintains the statistical power to detect socioeconomic differences that we would have lost had we undertaken subgroup analysis.

Further, we cannot exclude a level of correlation between these domains, but this is a limitation that we had to contend with when conceptualising distinct early-life domains and we discuss the formation of these more in our previous papers [16, 31]. It is likely—as shown by our modelling and previous evidence—that the effect of early life socioeconomic factors on MLTCs are mediated by adult factors including health behaviours [60], education [61] and own socioeconomic status [62]. In future research, we wish to further explore the role of this domain for MLTCs and health outcomes across the life course. However, it is important to highlight some of the difficulties we faced in relation to the existing evidence based on the effectiveness of relevant interventions. We found that most economic interventions often either focussed on a very specific subset of the population or provided a very general description of the potential economic impact, therefore making it difficult to directly map onto individual variables.

Finally, research that brings together multiple domains of early-life risk and real-world intervention effects to illustrate how such modelling might inform population-level prevention strategies, to provide a better reflection of real-world scenarios is lacking. Our paper provides one approach to address this. We recognise that given our modelling is hypothetical there is some level of uncertainty, and we encourage readers to interpret the results with caution. Our intention was not to present a definitive framework, but rather to contribute to the ongoing development of methods that better reflect the complexity of early life experiences and their long-term health impacts. The approach presented in this paper is just one way to address this complex question and there are likely to be different modelling approaches that should be explored and compared in future research. However, we see this work as one step in a broader effort to advance equity-focused, life course research. We hope it stimulates further discussion and methodological innovation in understanding how early-life prevention shapes health trajectories.

### Strengths and limitations

Data from a large cohort study provided some of the richest and most in-depth data in Britain and allowed us to capture a wide array of biological, social, environmental, behavioural and family variables in childhood to represent five early lifecourse domains. This depth of information would not have been available from most routinely collected electronic health care records in either primary or secondary care. The data also afforded the opportunity to consider a combined MLTC and burden outcome.

However, the cohort is representative of births occurring in Britain in 1970 and as such lacks ethnic diversity. We were also limited by the use of self-reported, rather than clinically measured, indicators of LTCs in adulthood, and by the specific conditions included in the age 46 data sweep. As a result, several common conditions were not available for analysis, including, but not limited to, chronic obstructive pulmonary disease, irritable bowel syndrome, chronic pain, attention deficit hyperactivity disorder, autism and osteoporosis, whilst using broad groupings of conditions has meant there is the possibility some conditions could have been misclassified. Additionally, the small sample size of participants with any one condition precluded the opportunity to investigate subgroup analysis, exploring how different clusters of conditions may have mediated the relationships considered differently. This is particularly important given that some LTCs are likely to be more burdensome than others for patients in terms of symptoms, impacting self-management demands (burden of treatment) and health-related quality of life [63–67].

Further our modelling approach assumed that effect estimates from interventions can be validly applied to a historical cohort. However, the populations from which these estimates were derived may differ from the BCS70 cohort in terms of calendar time, policy context and demographic characteristics. While we focused on comparable high-risk groups within the cohort, we acknowledge that the magnitude and nature of intervention effects may not translate directly across these contexts. This assumption introduces uncertainty into our estimates and could limit the generalisability of our findings. We have therefore interpreted the results as suggestive rather than definitive, and caution against overextending the implications beyond the scope of the hypothetical modelling framework. We also encourage further research to explore using such approaches using other cohorts and intervention estimates. However, we hope that by demonstrating this approach other researchers will be able to build on this methodology to further evaluate interventions in a way that can be then used to estimate their long-term impacts.

To avoid misclassification and ensure a clean comparison group, we excluded individuals who reported either role limitation without MLTCs or MLTCs without role limitation. This approach allowed us to isolate the group experiencing both exposures of interest—MLTCs and associated limitations—while ensuring the comparison group was free from either. Including individuals with only one component of the outcome would have introduced heterogeneity and risked conflating distinct trajectories of health and functioning. Future research could explore these subgroups using multinomial, latent class models or interaction terms, but this was beyond the scope of the current study.

When calculating domain adversity scores we assumed that each variable carried an equal relationship to the outcome, whereas in reality certain variables may have been more detrimental for the outcome compared to others. Additionally, by deriving variables into a binary indicator, we have potentially disregarded information contained within the original data structure, and in some instances, we were required to implement arbitrary cut-off points. For some of these variables where no standardised cut-off existed in the literature, we applied a pragmatic approach by using the bottom 10% of the distribution within the cohort to define adversity. This decision was based on the need to identify individuals who were meaningfully disadvantaged relative to the cohort; however, we knowledge the potential for misclassification and the need for caution when interpreting these thresholds. Further due to data limitations, we were only able to capture one aspect of burden (role limitations); however, a recent qualitative evidence synthesis identified eight themes of ‘work’ burden for those living with MLTCs [17], and it is important these themes of ‘work’ burden and incorporate into further research.

We found that the evidence of effectiveness on policy interventions with quantifiable statistics based on evaluation studies that could be incorporated into our prevention modelling was scarce. Thus, some of the policy interventions statistics were from populations outside the UK including the USA and Netherlands. Further, given effect estimates were derived from isolated studies, we were unable to capture the covariance between effect estimates, and it is anticipated that combinations of interventions will likely result in either a synergistic or sub-additive effect on the outcome that we could not consider. Further, we acknowledge that applying the same effect size across the full cohort could suggest a uniform benefit; however, our interpretation and conclusions are centred on the higher-risk subgroups. For example, we are not making inferences about the impact of the interventions on individuals in the zero adversity group, and we do not assume that these individuals would experience the same level of benefit given that their adversity score would not be able to be reduced. Further assuming a mean intervention effect across the cohort may not fully capture the heterogeneity of intervention responses. However, our approach was designed to provide a pragmatic estimate of potential impact using available effect sizes from real-world evaluations, which are based on average treatment effects. Future modelling could build on the methods presented here to incorporate stratified or individual-level effect estimates where available.

Finally, our PAF estimates presented apply specifically to the subgroup with MLTCs and role limitations within the BCS70 cohort, and may not generalise to the broader population. However, we believe this targeted approach is justified given our focus on identifying actionable early-life exposures that contribute to more severe health outcomes and informing discussions around the long-term important of early life interventions. This approach is also supported by Ferguson and O’Connell [68] who note that PAFs can be validly estimated in subpopulations, provided that causal exchangeability conditions are met and the exposure precedes the outcome.

## Conclusions

Early-life characteristics may play a role in the development of MLTCs with role limitations, particularly among individuals who experienced adversity during the prenatal, antenatal, neonatal, birth or early behavioural and developmental periods. This study presents a hypothetical modelling approach to explore what could potentially have happened if recent interventions targeting these domains had been implemented during childhood using the study sample of the BCS70 cohort members. Our findings suggest that such interventions could have led to a modest reduction in the risk of MLTCs with role limitations by age 46, especially among those exposed to early-life adversity in the prenatal, antenatal, neonatal and birth domain. These estimates are illustrative and intended to encourage further research in this area and inform policy discussions around assessing the impact of such interventions.

## Supplementary Information

Additional files 1. S1: SF-36 role limitations variables. Table S2. Variables included within each domain adversity scores. Table S3. Variables included in multivariable regression models. Table S4. The odds ratios of MLTCs with role limitation for parental-family environment adversity scores. Table S5. The odds ratios of MLTCs with role limitation for education and academic ability adversity scores. Table S6. The odds ratios of MLTCs with role limitation for prenatal, antenatal and neonatal and birth adversity scores. Table S7. The odds ratios of MLTCs with role limitation for the development and behaviour adversity scores. Table S8. The odds ratios of MLTCs with role limitation for socioeconomic factors adversity scores. Table S9. The odds ratios of MLTCs with role limitation for five childhood domains adversity scores. Figure S10. Changes in adversity scores and subsequent absolute reduction in risk of MLTCs with role limitation, for the significant PAF scenario of moving from a score of 3 + to 0 within the development and behaviour domain. Figure S11. Changes in adversity scores and subsequent absolute reduction in risk of MLTCs with role limitation, for the significant PAF scenario of moving from a score of 2 to 0 within the development and behaviour domain. Figure S12. Changes in adversity scores and subsequent absolute reduction in risk of MLTCs with role limitation, for significant PAF scenarios of moving from 3 + to 0 within the prenatal, antenatal, neonatal and birth domain.

## Data Availability

The data that support the findings of this study are available from UK Data Service. Restrictions apply to the availability of these data, which were used under license for this study. Data are available from https://ukdataservice.ac.uk/ with the permission of UK Data Service.

## Abbreviations

ACEs: Adverse Childhood Experiences
AUDIT-PC: Alcohol Use Disorders Identification Test for Primary Care
BCS70: 1970 British Cohort Study
BMI: Body Mass Index
FNP: Family Nurse partnership
LCHD: Life Course Health Development
LTCs: Long-term conditions
MELD-B: Multidisciplinary Ecosystem to study Lifecourse Determinants and Prevention of Early-onset Burdensome Multimorbidity
MLTCs: Multiple long-term conditions
OR: Odds ratio
PAFs: Population attributable fractions
UK: United Kingdom
USA: United States of America
