# Supplementary material for: Modelling the joint impact of early-life interventions on adult health: an illustrative example of multiple long-term conditions with role limitations in midlife using the 1970 British Cohort Study (BCS70)
Source: BMC Med. 2025 Nov 18;23:642. doi: 10.1186/s12916-025-04467-3 (PMC12625343; doi:10.1186/s12916-025-04467-3)
Supplement: Supplementary file 1 — Additional files 1. S1: SF-36 role limitations variables. Table S2. Variables included within each domain adversity scores. Table S3. Variables included in multivariable regression models. Table S4. The odds ratios of MLTCs with role limitation for parental-family environment adversity scores. Table S5. The odds ratios of MLTCs with role limitation for education and academic ability adversity scores. Table S6. The odds ratios of MLTCs with role limitation for prenatal, antenatal and neonatal and birth adversity scores. Table S7. The odds ratios of MLTCs with role limitation for the development and behaviour adversity scores. Table S8. The odds ratios of MLTCs with role limitation for socioeconomic factors adversity scores. Table S9. The odds ratios of MLTCs with role limitation for five childhood domains adversity scores. Figure S10. Changes in adversity scores and subsequent absolute reduction in risk of MLTCs with role limitation, for the significant PAF scenario of moving from a score of 3 + to 0 within the development and behaviour domain. Figure S11. Changes in adversity scores and subsequent absolute reduction in risk of MLTCs with role limitation, for the significant PAF scenario of moving from a score of 2 to 0 within the development and behaviour domain. Figure S12. Changes in adversity scores and subsequent absolute reduction in risk of MLTCs with role limitation, for significant PAF scenarios of moving from 3 + to 0 within the prenatal, antenatal, neonatal and birth domain. [file 12916_2025_4467_MOESM1_ESM.docx]

**Exploring the Relationship Between Early Life Exposures and the Odds of Multimorbidity with Role Limitations: Findings from the 1970 The British Cohort Study (BCS70)**

Supplementary Materials

*S1. SF-36 role limitations variables*

SF-36 Role-limitations due to physical health

Physical health score coded on 4 items. Lower scores indicate greater lifestyles limitations as a result of physical health problems in the four week prior to interview. All four items are coded the same. Responses of ‘Yes’ score 0, as health has limited the cohort member (1=0) while ‘No’ is 100 (2=100). Scores are the mean of the total number of questions answered (1-4 responses).

4 items:

1. Have you cut down the amount of time you spent on work or other activities
2. Have you accomplished less than you would like
3. Were limited in the kind of work or other activities
4. Had difficulty performing the work or other activities (for example, it took extra effort)

SF-36 Role-limitations due to emotional health

Physical health score coded on 3 items. Lower scores indicate greater lifestyles limitations as a result of emotional problems in the four week prior to interview. All three items are coded the same. Responses of ‘Yes’ score 0, as health has limited the cohort member (1=0) while ‘No’ is 100 (2=100). Scores are the mean of the total number of questions answered (1-3 responses).

3 items:

1. Have you cut down the amount of time you spent on work or other activities
2. Have you accomplished less than you would like
3. Didn’t do work or other activities as careful as usual

*Table S2. Variables included within each domain adversity scores.*

|  | 0 score (no adversity) | 1 score (adversity) |
| --- | --- | --- |
| **Antenatal, Neonatal, Prenatal and Birth Domain** | | |
| Maternal age at cohort members birth | *Oldest 90% of all mothers* | *Youngest 10% of all mothers* |
| Parity | *3 or lower* | *4 or higher* |
| Ever a teenage mother | *No* | *Yes* |
| Maternal smoking during pregnancy | *Non-smoker* | *Smoked during pregnancy* |
| Birthweight | *Over 2500g* | *Less than 2500g* |
| **Developmental Attributes and Behaviour Domain** | | |
| Number of steps when walking backwards | *Highest 90% of scores* | *Lowest 10% of scores* |
| Balance when standing on right leg | *Steady* | *Unsteady* |
| Balance when standing on left leg | *Steady* | *Unsteady* |
| Difficulty when kicking a ball | *Highest 90% of scores* | *Lowest 10% of scores* |
| Poor hand control | *Highest 90% of scores* | *Lowest 10% of scores* |
| Difficulty when picking up objects | *Highest 90% of scores* | *Lowest 10% of scores* |
| Does child display outbursts of temper | *Highest 90% of scores* | *Lowest 10% of scores* |
| Rutter behaviour^1^ | *Normal behaviour* | *Moderate or severe behavioural* |
| Emotional or behavioural problems | *No* | *Yes* |
| **Socioeconomic Factors Domain** | | |
| Parental occupational social class^2^ | *I, II, III* | *V, IV* |
| Parental van/car ownership | *Yes* | *No* |
| Cohort member lives on a council estate | *No* | *Yes* |
| Income | *Over £49 per week* | *Under £49 per week* |
| Housing tenure | *Owner/private rent* | *Council rent/other* |
| Father employment | *Employed* | *Unemployed/no father* |
| **Education and Academic Ability Domain** | | |
| Edinburgh reading test score | *Highest 90% of scores* | *Lowest 10% of scores* |
| Friendly math test score | *Highest 90% of scores* | *Lowest 10% of scores* |
| Difficult reading – maternal rating | *No difficulty* | *Some difficulty* |
| Difficult maths – maternal rating | *No difficulty* | *Some difficulty* |
| Difficult spelling – maternal rating | *No difficulty* | *Some difficulty* |
| Ability reading – cohort member rating | *Good ability* | *Poor ability* |
| Ability math – cohort member rating | *Good ability* | *Poor ability* |
| Ability spelling – cohort member rating | *Good ability* | *Poor ability* |
| **Parental-Family Environment Domain** | | |
| Family go on walks | *Sometimes or often* | *Rarely or never* |
| Family go on outings | *Sometimes or often* | *Rarely or never* |
| Family has meals together | *Sometimes or often* | *Rarely or never* |
| Family goes on holidays | *Sometimes or often* | *Rarely or never* |
| Family goes shopping | *Sometimes or often* | *Rarely or never* |
| Family goes to restaurants | *Sometimes or often* | *Rarely or never* |
| Father helps manage child | *Yes* | *No/No father* |
| Mother interested in child education – teacher rating | *Some interest* | *Little/No interest* |

*^1^Total Score for Rutter behaviour scale, a scale that provides an indication of behaviour difficulties.*

*^2^Father social classed used in the first instant, but mother social class used is father was absent.*

*Table S3. Variables included in multivariable regression models and the reporting of MLTCs with role limitations at age 46. The total sample size was based on the specific sample available at the sweep in which the variable was reported or measured.*

| Variable |  | Number (%) with MLTCs with role limitations | Total sample |
| --- | --- | --- | --- |
| Sex | Men | 234  (8.65%) | 2705 |
|  | Women | 399  (15.06%) | 2649 |
| Ethnicity | English | 564  (11.66%) | 4839 |
|  | Other | 26  (12.21%) | 213 |
| Number of days of exercise per week | 0 | 319  (23.03%) | 1385 |
|  | 1 | 47  (7.97%) | 590 |
|  | 2 | 71  (8.95%) | 793 |
|  | 3 | 81  (9.06%) | 894 |
|  | 4 | 28  (5.19%) | 539 |
|  | 5 | 57  (8.12%) | 702 |
|  | 6 | 16  (7.27%) | 220 |
|  | 7 | 103  (10.16%) | 1014 |
| Highest educational qualification | No qualification | 279  (16.16%) | 1726 |
|  | GCSE | 220  (11.45%) | 1922 |
|  | A or AS level | 32  (9.17%) | 349 |
|  | Diploma | 63  (12.30%) | 512 |
|  | Degree | 104  (8.16%) | 1274 |
|  | Higher degree | 25  (7.58%) | 330 |
| Smoking status | Non-smoker | 489  (9.83%) | 4974 |
|  | Smoker | 247  (20.15%) | 1226 |
| Occupational social class | 1 | 46  (4.50%) | 1016 |
|  | 2 | 135  (7.97%) | 1694 |
|  | 3 | 67  (10.36%) | 647 |
|  | 4 | 38  (7.50%) | 507 |
|  | 5 | 37  (7.96%) | 465 |
|  | 6 | 46  (9.62%) | 478 |
|  | 7 | 31  (8.54%) | 363 |
|  | Not stated | 336  (32.62%) | 103 |
| Hours spent watching TV | 0 | 87  (9.36%) | 929 |
|  | 1 | 184  (8.91%) | 2066 |
|  | 2 | 169  (10.19%) | 1658 |
|  | 3 | 138  (17.62%) | 883 |
|  | 4+ | 152  (35.43%) | 429 |
| Hours spent on the Internet | 0 | 238  (10.54%) | 2257 |
|  | 1 | 239  (10.41%) | 2295 |
|  | 2 | 107  (15.42%) | 694 |
|  | 3 | 47  (17.28%) | 272 |
|  | 4+ | 98  (27.68%) | 354 |
| Cohabiting with partner | Yes | 416  (8.79%) | 4731 |
|  | No | 320  (21.78%) | 1469 |
| IMD quintiles | 1 | 91  (24.20%) | 376 |
|  | 2 | 81  (21.32%) | 380 |
|  | 3 | 81  (17.23%) | 470 |
|  | 4 | 80  (15.30%) | 523 |
|  | 5 | 72  (11.75%) | 613 |
|  | 6 | 76  (11.05%) | 688 |
|  | 7 | 67  (9.60%) | 698 |
|  | 8 | 74  (9.50%) | 779 |
|  | 9 | 51  (6.57%) | 776 |
|  | 10 | 61  (6.93%) | 880 |
| Financial difficulty | Comfortable | 122  (5.11%) | 2387 |
|  | Doing okay | 245  (9.46%) | 2589 |
|  | Just about getting by | 213  (23.54%) | 905 |
|  | Finding it difficult | 81  (39.90%) | 203 |
|  | Finding it very difficulty | 74  (66.67%) | 111 |
| AUDIT-PC classification | No alcohol consumption | 181  (27.14%) | 667 |
|  | Unproblematic | 355  (8.66%) | 4101 |
|  | Problematic | 189  (13.31%) | 1420 |
| Weekly income – No MLTCs with role limitation: mean (SD) | | £994.00 (£2175.68) | 4981 |
| Weekly income – MLTCs with role limitation mean (SD) | | £548.45 (£932.47) | 673 |

| N=6201  (50 imputations) |  | Model 1: Adjusting for sex and ethnicity | | Model 2: Adjusting for sex and ethnicity and socioeconomic factors | | Model 3: Adjusting for sex and ethnicity and all other domains | |
| --- | --- | --- | --- | --- | --- | --- | --- |
|  | Score | OR | 95% CI | OR | 95% CI | OR | 95% CI |
| Parental-family environment score – ref: 0 | 1 | **1.36** | **1.13 - 1.65** | **1.24** | **1.02 - 1.51** | **1.23** | **1.01 - 1.49** |
|  | 2 | **1.42** | **1.10 - 1.86** | 1.17 | 0.89 - 1.54 | 1.09 | 0.82 - 1.44 |
|  | 3+ | **2.16** | **1.63 - 2.86** | **1.65** | **1.23 - 2.22** | **1.49** | **1.10 - 2.03** |

*Table S4. The odds ratios of MLTCs with role limitation for parental-family environment adversity scores. Adjusting for sex and ethnicity (model 1), the socioeconomic domain (model 2), and the other domains (model 3).*

*Statistically significant figures using a 95% level are included in bold.*

*Table S5. The odds ratios of MLTCs with role limitation for education and academic ability adversity scores. Adjusting for sex and ethnicity (model 1), the socioeconomic domain (model 2), and the other domains (model 3).*

| N=6201  (50 imputations) |  | Model 1: Adjusting for sex and ethnicity | | Model 2: Adjusting for sex and ethnicity and socioeconomic factors | | Model 3: Adjusting for sex and ethnicity and all other domains | |
| --- | --- | --- | --- | --- | --- | --- | --- |
|  | Score | OR | 95% CI | OR | 95% CI | OR | 95% CI |
| Education and academic ability score – ref: 0 | 1 | **1.30** | **1.03 - 1.66** | **1.28** | **1.00 - 1.62** | 1.22 | 0.96 - 1.56 |
|  | 2 | **1.51** | **1.16 - 1.97** | **1.43** | **1.10 - 1.54** | **1.31** | **1.01 - 1.71** |
|  | 3+ | **1.85** | **1.43 - 2.39** | **1.63** | **1.26 - 2.12** | **1.31** | **1.00 - 1.72** |

*Statistically significant figures using a 95% level are included in bold.*

*Table S6. The odds ratios of MLTCs with role limitation for prenatal, antenatal and neonatal and birth adversity scores. Adjusting for sex and ethnicity (model 1), the socioeconomic domain (model 2), and the other domains (model 3).*

| N=6201  (50 imputations) |  | Model 1: Adjusting for sex and ethnicity | | Model 2: Adjusting for sex and ethnicity and socioeconomic factors | | Model 3: Adjusting for sex and ethnicity and all other domains | |
| --- | --- | --- | --- | --- | --- | --- | --- |
|  | Score | OR | 95% CI | OR | 95% CI | OR | 95% CI |
| Prenatal, antenatal, neonatal and birth score – ref: 0 | 1 | **1.49** | **1.22 - 1.82** | **1.36** | **1.10 - 1.67** | **1.33** | **1.08 - 1.63** |
|  | 2 | **1.66** | **1.31 - 2.12** | **1.38** | **1.07 - 1.78** | 1.27 | 0.97 - 1.64 |
|  | 3+ | **2.83** | **2.10 - 3.81** | **2.20** | **1.60 - 3.03** | **2.01** | **1.45 - 2.78** |

*Statistically significant figures using a 95% level are included in bold.*

*Table S7. The odds ratios of MLTCs with role limitation for the development and behaviour adversity scores. Adjusting for sex and ethnicity (model 1), the socioeconomic domain (model 2), and the other domains (model 3).*

| N=6201  (50 imputations) |  | Model 1: Adjusting for sex and ethnicity | | Model 2: Adjusting for sex and ethnicity and socioeconomic factors | | Model 3: Adjusting for sex and ethnicity and all other domains | |
| --- | --- | --- | --- | --- | --- | --- | --- |
|  | Score | OR | 95% CI | OR | 95% CI | OR | 95% CI |
| Development and behaviour domain score – ref: 0 | 1 | **1.29** | **1.01 - 1.66** | 1.25 | 0.98 - 1.60 | 1.22 | 0.95 - 1.56 |
|  | 2 | **1.56** | **1.23 - 2.00** | **1.48** | **1.16 - 1.89** | **1.41** | **1.10 - 1.81** |
|  | 3+ | **2.44** | **1.93 - 3.09** | **2.24** | **1.76 - 2.86** | **2.09** | **1.63 - 2.67** |

*Statistically significant figures using a 95% level are included in bold.*

| N=6201  (50 imputations) |  | Model 1: Adjusting for sex and ethnicity | | Model 2: Adjusting for sex and ethnicity and development and behaviour domain | | Model 3: Adjusting for sex and ethnicity and  prenatal, antenatal, neonatal and birth domain | | Model 4: Adjusting for sex and ethnicity and education and academic ability domain | | Model 5: Adjusting for sex and ethnicity and parental-family environment domain | | Model 6: Adjusting for sex and ethnicity and all other domains | |
| --- | --- | --- | --- | --- | --- | --- | --- | --- | --- | --- | --- | --- | --- |
|  | Score | OR | 95% CI | OR | 95% CI | OR | 95% CI | OR | 95% CI | OR | 95% CI | OR | 95%CI |
| Socioeconomic factors domain – ref: 0 | 1 | **1.28** | **1.01 - 1.61** | 1.24 | 0.98 - 1.56 | 1.20 | 0.95 - 1.53 | 1.25 | 0.99-1.58 | 1.23 | 0.97-1.56 | 1.13 | 0.89 - 1.44 |
|  | 2 | **1.43** | **1.12 - 1.84** | **1.38** | **1.07 - 1.77** | 1.27 | 0.99 - 1.64 | **1.39** | **1.09-1.79** | **1.35** | **1.05-1.73** | 1.18 | 0.90 - 1.53 |
|  | 3+ | **2.20** | **1.76 - 2.75** | **2.03** | **1.61 – 2.55** | **1.83** | **1.43 - 2.34** | **2.08** | **1.66-2.61** | **1.96** | **1.54-2.49** | **1.55** | **1.19 - 2.02** |

*Table S8. The odds ratios of MLTCs with role limitation for socioeconomic factors adversity scores. Adjusting for sex and ethnicity (model 1), the development and behaviour domain (model 2), the prenatal, antenatal, neonatal and birth domain (model 3), the education and academic ability domain (model 4), the parental-family environment domain (model 5) and all the other domains combined (model 6).*

*Statistically significant figures using a 95% level are included in bold.*

*Table S9. The odds ratios of MLTCs with role limitation for five childhood domains adversity scores. Adjusting for sex and ethnicity, the other domains, and adult factors.*

|  | Odds ratio | 95% confidence  interval | |
| --- | --- | --- | --- |
| Parental-family environment score – Ref: 0 |  |  |  |
| 1 | 1.19 | 0.96 | 1.49 |
| 2 | 0.89 | 0.64 | 1.25 |
| 3 | 1.25 | 0.88 | 1.80 |
| Prenatal, antenatal, neonatal and birth score – Ref: 0 |  |  |  |
| 1 | 1.24 | 0.98 | 1.58 |
| 2 | 1.10 | 0.81 | 1.49 |
| 3 | **1.70** | **1.14** | **2.52** |
| Development and behaviour domain score – Ref: 0 |  |  |  |
| 1 | 1.14 | 0.87 | 1.49 |
| 2 | 1.32 | 1.00 | 1.75 |
| 3 | **1.65** | **1.25** | **2.19** |
| Education and academic ability score – Ref: 0 |  |  |  |
| 1 | 1.12 | 0.85 | 1.47 |
| 2 | 1.04 | 0.76 | 1.41 |
| 3 | 0.89 | 0.64 | 1.23 |
| Socioeconomic factors score – Ref: 0 |  |  |  |
| 1 | 1.01 | 0.76 | 1.34 |
| 2 | 0.94 | 0.68 | 1.30 |
| 3 | 0.92 | 0.67 | 1.28 |
| Mother ethnicity - Ref: white |  |  |  |
| Other | 0.67 | 0.39 | 1.15 |
| Sex – Ref: male |  |  |  |
| Female | **1.81** | **1.46** | **2.23** |
| Educational qualification – Ref: no qualification |  |  |  |
| GCSE | 1.00 | 0.79 | 1.26 |
| A/AS level | 0.96 | 0.61 | 1.52 |
| Diploma | 1.25 | 0.88 | 1.78 |
| Degree | 1.19 | 0.87 | 1.62 |
| Higher Degree | 1.32 | 0.79 | 2.18 |
| Exercise per week – Ref: 0 days |  |  |  |
| 1 day | **0.51** | **0.35** | **0.74** |
| 2 days | **0.54** | **0.39** | **0.74** |
| 3 days | **0.62** | **0.46** | **0.83** |
| 4 days | **0.34** | **0.22** | **0.52** |
| 5 days | **0.53** | **0.38** | **0.74** |
| 6 days | **0.46** | **0.26** | **0.84** |
| 7 days | **0.49** | **0.37** | **0.65** |
| Number of hours on the internet per day – Ref: 0-1 days |  |  |  |
| 1 to 2 hours a day | 1.08 | 0.87 | 1.36 |
| 2 to 3 hours a day | **1.42** | **1.06** | **1.89** |
| 3 to 4 hours a day | 1.03 | 0.69 | 1.56 |
| More than 4 hours a day | **1.82** | **1.29** | **2.56** |
| Number of hours watching tv per day – Ref: 0-1 days |  |  |  |
| 1 to 2 hours a day | 1.09 | 0.80 | 1.48 |
| 2 to 3 hours a day | 1.06 | 0.77 | 1.46 |
| 3 to 4 hours a day | **1.61** | **1.14** | **2.27** |
| More than 4 hours a day | **2.16** | **1.48** | **3.17** |
| Occupational social class – Ref: higher professional |  |  |  |
| Administrative and professional occupations | 1.37 | 0.95 | 1.98 |
| Intermediate occupations | 1.26 | 0.82 | 1.95 |
| Small employers and own account workers | 1.07 | 0.66 | 1.74 |
| Lower supervisory and technical occupations | 1.13 | 0.69 | 1.86 |
| Semi-routine occupations | 0.91 | 0.56 | 1.47 |
| Routine occupations | 0.86 | 0.50 | 1.47 |
| Occupation Not Stated | **2.51** | **1.68** | **3.73** |
| Not Applicable | **5.71** | **3.76** | **8.67** |
| Partnership status- Ref: cohabiting with partner |  |  |  |
| No partnership | **1.62** | **1.32** | **1.98** |
| Alcohol consumption – Ref: does not drink |  |  |  |
| Unproblematic drinking (0-4) | **2.21** | **1.71** | **2.85** |
| Increasing or higher risk drinking (5+) | **1.53** | **1.22** | **1.92** |
| Smoking status – Ref: non smoker |  |  |  |
| Smoker | **1.26** | **1.02** | **1.57** |
| Weekly income | 1.00 | 1.00 | 1.00 |
| Self-reported financial status – Ref: Comfortable |  |  |  |
| Doing all right | **1.56** | **1.22** | **1.99** |
| Just about getting by | **3.20** | **2.43** | **4.21** |
| Finding it quite difficult | **6.72** | **4.54** | **9.95** |
| Finding it very difficult | **14.07** | **8.40** | **23.57** |
| IMD quintile – Ref: 1 - most deprived |  |  |  |
| 2 | 1.26 | 0.82 | 1.91 |
| 3 | 0.96 | 0.63 | 1.46 |
| 4 | 0.97 | 0.64 | 1.46 |
| 5 | 1.01 | 0.66 | 1.53 |
| 6 | 0.97 | 0.64 | 1.47 |
| 7 | 0.93 | 0.61 | 1.42 |
| 8 | 0.92 | 0.61 | 1.40 |
| 9 | **0.61** | **0.39** | **0.96** |
| 10 - least deprived decile | 0.81 | 0.52 | 1.24 |

*Statistically significant figures using a 95% level are included in bold.*

*
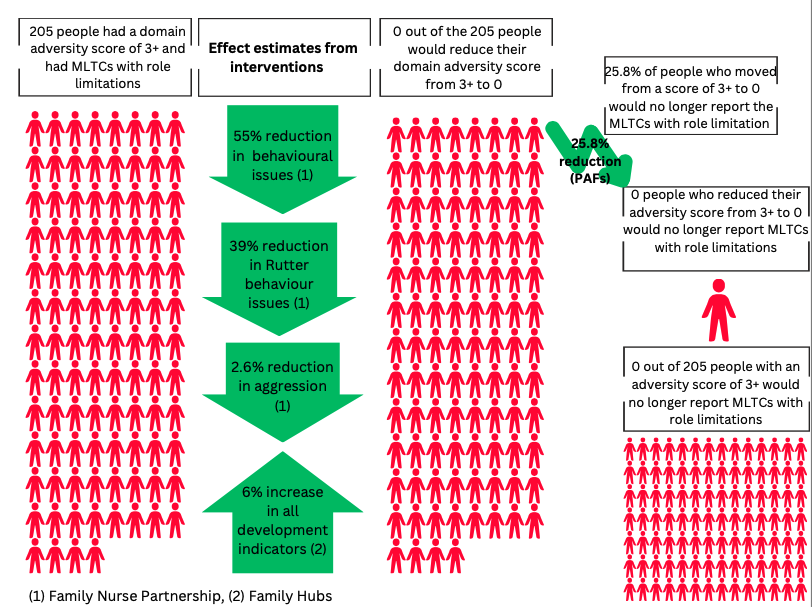
Figure S.10. Modelling the fully adjusted changes in adversity scores and subsequent absolute reduction in risk of MLTCs* *with role limitation, for the significant PAF scenario of moving from a score of 3+ to 0 within the development and behaviour domain.*

*Figure S.11. Modelling the fully adjusted changes in adversity scores and subsequent absolute reduction in risk of MLTCs* *with role limitation, for the significant PAF scenario of moving from a score of 2 to 0 within the development and behaviour domain.*

*
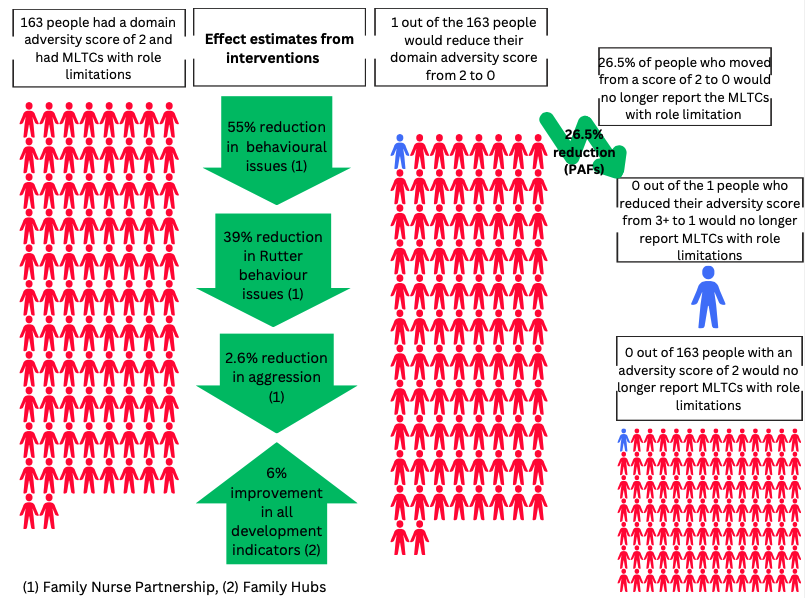
*

*Figure S12. Modelling the fully adjusted changes in adversity scores and subsequent absolute reduction in risk of MLTC*s *with role limitation, for significant PAF scenarios of moving from 3+ to 0 within the* *prenatal, antenatal, neonatal and birth domain.*

*
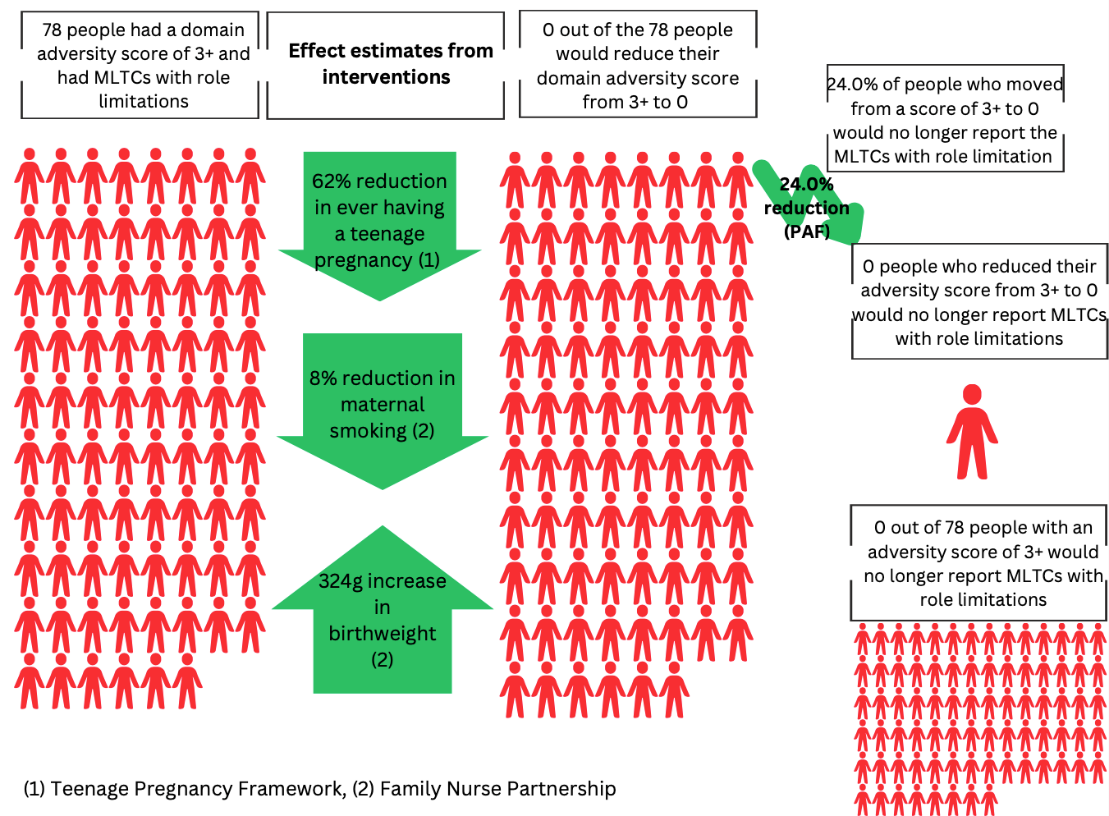
*
